# Defining distal splenopancreatectomy by the mesopancreas

**DOI:** 10.1007/s00423-024-03320-0

**Published:** 2024-04-16

**Authors:** S.-A. Safi, A. Alexander, W. Neuhuber, L. Haeberle, A. Rehders, T. Luedde, I. Esposito, G. Fluegen, W. T. Knoefel

**Affiliations:** 1 Departments of Surgery (A), Heinrich-Heine-University and University Hospital Duesseldorf, Moorenstr. 5, 40225 Duesseldorf, Germany; 2 Institute of Anatomy I, Friedrich-Alexander University Erlangen-Nuremberg, Universitätsstr. 1, Erlangen, Germany; 3 Institute of Pathology, Heinrich-Heine-University and University Hospital Duesseldorf, Moorenstr. 5, 40225 Duesseldorf, Germany; 4 Department of Gastroenterology, Hepatology and Infectious Diseases, Heinrich-Heine-University and University Hospital Duesseldorf, Moorenstr. 5, 40225 Duesseldorf, Germany

**Keywords:** PDAC, Ductal adenocarcinoma of the pancreas, Pancreatic cancer, CRM, Mesopancreatic excision, Survival outcome, Peripancreatic tissue, Distal pancreatectomy

## Abstract

**Background:**

The implementation of the pathologic CRM (circumferential resection margin) staging system for pancreatic head ductal adenocarcinomas (hPDAC) resulted in a dramatic increase of R1 resections at the dorsal resection margin, presumably because of the high rate of mesopancreatic fat (MP) infiltration. Therefore, mesopancreatic excision (MPE) during pancreatoduodenectomy has recently been promoted and has demonstrated better local disease control, fueling the discussion of neoadjuvant downsizing regimes in MP + patients. However, it is unknown to what extent the MP is infiltrated in patients with distal pancreatic (tail/body) carcinomas (dPDAC). It is also unknown if the MP infiltration status affects surgical margin control in distal pancreatectomy (DP). The aim of our study was to histopathologically analyze MP infiltration and elucidate the influence of resection margin clearance on recurrence and survival in patients with dPDAC. Furthermore, the results were compared to a collective receiving MPE for hPDAC.

**Method:**

Clinicopathological and survival parameters of 295 consecutive patients who underwent surgery for PDAC (*n* = 63 dPDAC and *n* = 232 hPDAC) were evaluated. The CRM evaluation was performed in a standardized fashion and the specimens were examined according to the Leeds pathology protocol (LEEPP). The MP area was histopathologically evaluated for cancerous infiltration.

**Results:**

In 75.4% of dPDAC patients the MP fat was infiltrated by vital tumor cells. The rates of MP infiltration and R0CRM– resections were similar between dPDAC and hPDAC patients (*p* = *0.497 and 0.453 respectively*). MP– infiltration status did not correlate with CRM implemented resection status in dPDAC patients (*p* = *0.348*). In overall survival analysis, resection status and MP status remained prognostic factors for survival. In follow up analysis. surgical margin clearance in dPDAC patients was associated with a significant improvement in local recurrence rates (5.2% in R0CRM– resected vs. 33.3 in R1/R0CRM + resected, *p* = *0.002*).

**Conclusion:**

While resection margin status was not affected by the MP status in dPDAC patients, the high MP infiltration rate, as well as improved survival in MP– dPDAC patients after R0CRM– resection, justify mesopancreatic excision during splenopancreatectomy. Larger scale studies are urgently needed to validate our results and to study the effect on neoadjuvant treatment in dPDAC patients.

## Introduction

Poor survival outcome in pancreatic cancer patients is partially due to late diagnosis and consequently, advanced tumor stage. Thus, it is estimated that only 20% of all diagnosed patients are eligible for surgical therapy up front [1]. Distal PDACs of the pancreatic tail/body (dPDAC) constitute about one third of all patients with exocrine pancreatic cancer [2]. Most of the studies on pathological and survival outcome, as well as on resection techniques, are focused on the more prevalent PDAC of the pancreatic head (hPDAC) [3].

The histopathological examination protocol for pancreatic head carcinomas was redefined in 2004 according to the recommendations of the Royal College of Pathologists (LEEPP) [3, 4]. By implementing the circumferential resection margin (CRM), this protocol allows a more detailed assessment of the resected specimen, as all resection margins are taken into account [5, 6]. While the rate of true margin negative resections has significantly dropped, the dorsal resection margin and the vascular groove remained the main site for insufficient tumor clearance [3]. We previously described the technique of mesopancreatic excision (MPE) during structured pancreatoduodenectomy for hPDAC, identifying a surprisingly high rate of infiltration of the mesopancreatic fat, which resembles the area covering the dorsal resection margin and thus the peripancreatic fat [7, 8].

Surgical resection using embryo-anatomic landmarks has been already implemented and standardized for other abdominal cancers [9, 10]. Mesorectal and mesocolic excisions, now standardly applied, have before endured a matter of debate, are utilized by fusion fascia, hence the implemented idea of compartment anatomy [10–12], strengthening the clinical relevance of the mesentery in secondary retroperitoneal organs.

While pancreatoduodenectomy is the single recognized resection technique for pancreatic head malignancies, the surgical approach in dPDAC is still heterogenous. While some authors prefer a minimal invasive, spleen preserving technique, others favor an extensive surgical approach by standardized splenopancreatectomy [13–16]. A uniform gold standard however does not exist.

Strasberg et al. introduced an antegrade approach to achieve margin clearance during radical splenopancreatectomy and described the utilization of the Gerota fascia for the posterior margin [17]. The study-line on the superiority of this method is heterogenous [18–20]. Since the embryologic rotation of the pancreatic body/tail result in secondary retropancreatic fusion fascia formation, in our opinion the idea of compartment anatomy can be translated to the distal peripancreatic region as well [12, 21]. Previous studies have not considered a possible infiltration of the mesopancreas as a stratification parameter for R-status which justifies the surgical approach proposed by Strasberg [20].

While the mesopancreatic (MP) fat surrounding the pancreatic head has been studied in detail [7, 22–26], it remains unknown if the mesopancreatic fat surrounding the pancreatic body and tail is equally affected in dPDAC patients undergoing curative resection. It also remains unknown if the mesopancreatic infiltration status affects surgical margin clearances. While LEEPP was implemented for all PDACs in our institution, the aim of this study was to evaluate the MP area in a consecutive cohort and compare this data with hPDAC patients undergoing pancreatoduodenectomy. To underline the clinical relevance of our findings, survival analysis and distribution analysis of metachronous disease in dPDAC patients was performed. During structured oncologic distal splenopancreatectomy, we routinely perform a radical antegrade modular pancreatosplenectomy (RAMPS), which is a fascia-oriented approach, as described by Strasberg et al. [7, 12, 18, 21, 25, 27]. Since the existence and oncological relevance of the peripancreatic fascial system remains a matter of some debate, the second aim of this study was to anatomically describe the posterior fascial covering of the distal pancreas by surgical dissection of body donors.

## Material and method

### Patient selection and demographic data

All PDAC patients who received MPE during pancreatoduodenectomy (hPDAC) or distal splenopancreatectomy (dPDAC) with curative intent at the University Hospital of Duesseldorf between 2015 and 2021, irrespective of tumor stage and microscopic resection margin, were included in this study from a prospectively maintained database. Inclusion criteria were: surgically resected PDAC without neoadjuvant therapy, available histopathological specimen and follow-up examinations with sufficient information on relapse status and location of relapse. Patients who underwent surgery for other malignant tumors were excluded from the study. TNM staging, grading, perineural invasion as well as lymphatic and venous invasion were obtained from the original pathological reports and if applicable updated to the 8th UICC edition [28]. Histopathological slides were re-visited by an experienced pathologist for pancreatic cancer (L.H.) with focus on mesopancreatic fat invasion and to re-evaluate the resection margin of cases prior to CRM protocol implementation [6]. Data regarding site of metastasis, were recorded. The study was carried out in accordance with the guidelines of Good Clinical Practice and the Declaration of Helsinki. The study was approved by the Institutional Review Board (IRB) of the Medical Faculty, Heinrich Heine University Duesseldorf (IRB-no. 2022–1990).

### Operative procedure

**Fig. 1:**
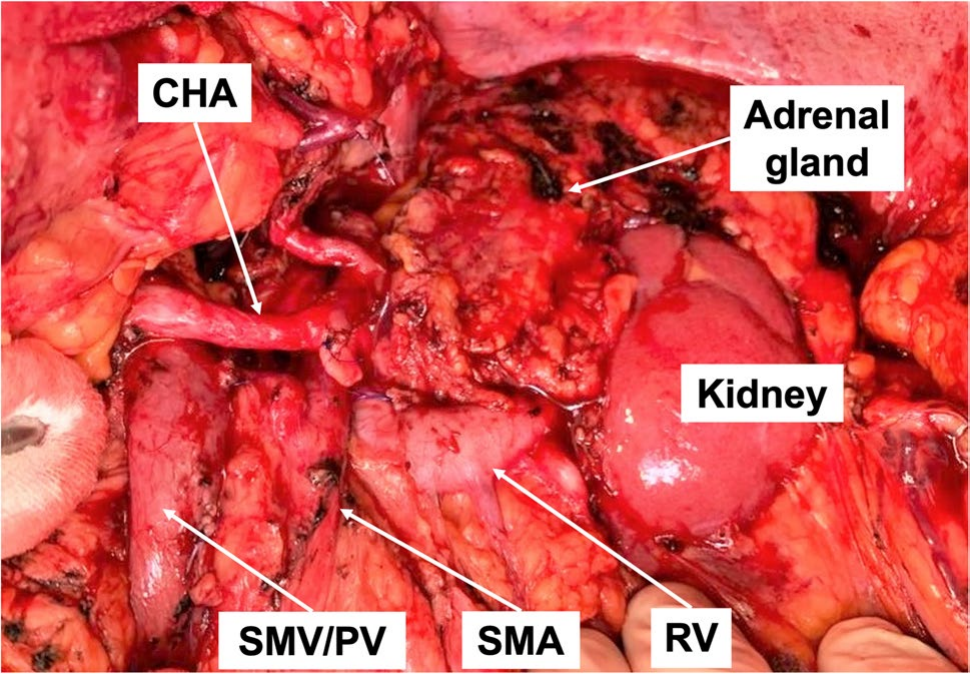
Intraoperative picture demonstrating the surgical site after structured splenopancreatectomy for dPDAC. Complete skeletonization of the SMA is only carried out for 180° of the left circumference. Only in selected cases in which tumor encasement is intraoperatively suspicious, an extended dissection > 180° of the SMA is carried out. AA: abdominal aorta; CHA: common hepatic artery; IVC: inferior vena cava; LRV: left renal vein; PV: portal vein; SMA: superior mesenteric artery

The principles of MPE during structured PD for hPDAC have already been described in detail [7, 18, 25]. Following the same principles, oncological distal splenopancreatectomy is routinely performed for dPDAC in our institution: After establishing a clear view of the duodenum, pancreas and spleen, the splenic artery (SA) is dissected close from the celiac trunk (CT). The transverse and descending colon are dissected from the Toldts fascia, as performed in mesocolic excision [10] in order to access the inferior border of the peripancreatic fat. Treitz’s ligament is divided and the SMV and SMA are presented and the ventral and left aspect of these vessels are dissected. This allows to visualize the inferior mesenteric vein (IMV) which is divided before entering the mesopancreas. Dissecting along the SMV, the splenic vein (SV) is identified and dissected close to its junction with the SMV. The pancreas is then divided on top of the mesenterico-portal axis. This line of transection needs to be adjusted to provide a safe distance to the pancreatic tumor. If a possible tumor infiltration is detected during these steps, venous resection and reconstruction is routinely performed. The gastrosplenic ligament is divided close to the greater curvature. Sharp preparations along the SMA and the CT up to their aortic origins is carried out. To avoid persistent diarrhea only 180° to 270° of the left circumference of the SMA are dissected. If cancerous involvement is intraoperatively suspected, dissection of the SMA is extended to the left circumference (Fig. 1). This allows an extensive retroperitoneal lymph node dissection to the left side of the aorta. The dissection is then continued from medial to lateral on the left adrenal and Gerota’s fascia, which is then carried out cranially, until mobilization of the spleen is complete.

In summary, the aim of the procedure is a complete dissection of perineural and lymphatic tissue and structures surrounding the pancreatic body and tail (CHA, CT, SMA, PV/SMV, SA and SV/IMV), in an “en bloc” resection (Fig. 1). Depending on the size and location of the tumor, the order of the above operative steps may have to be adapted.

### Anatomical preparations

**Fig. 2:**
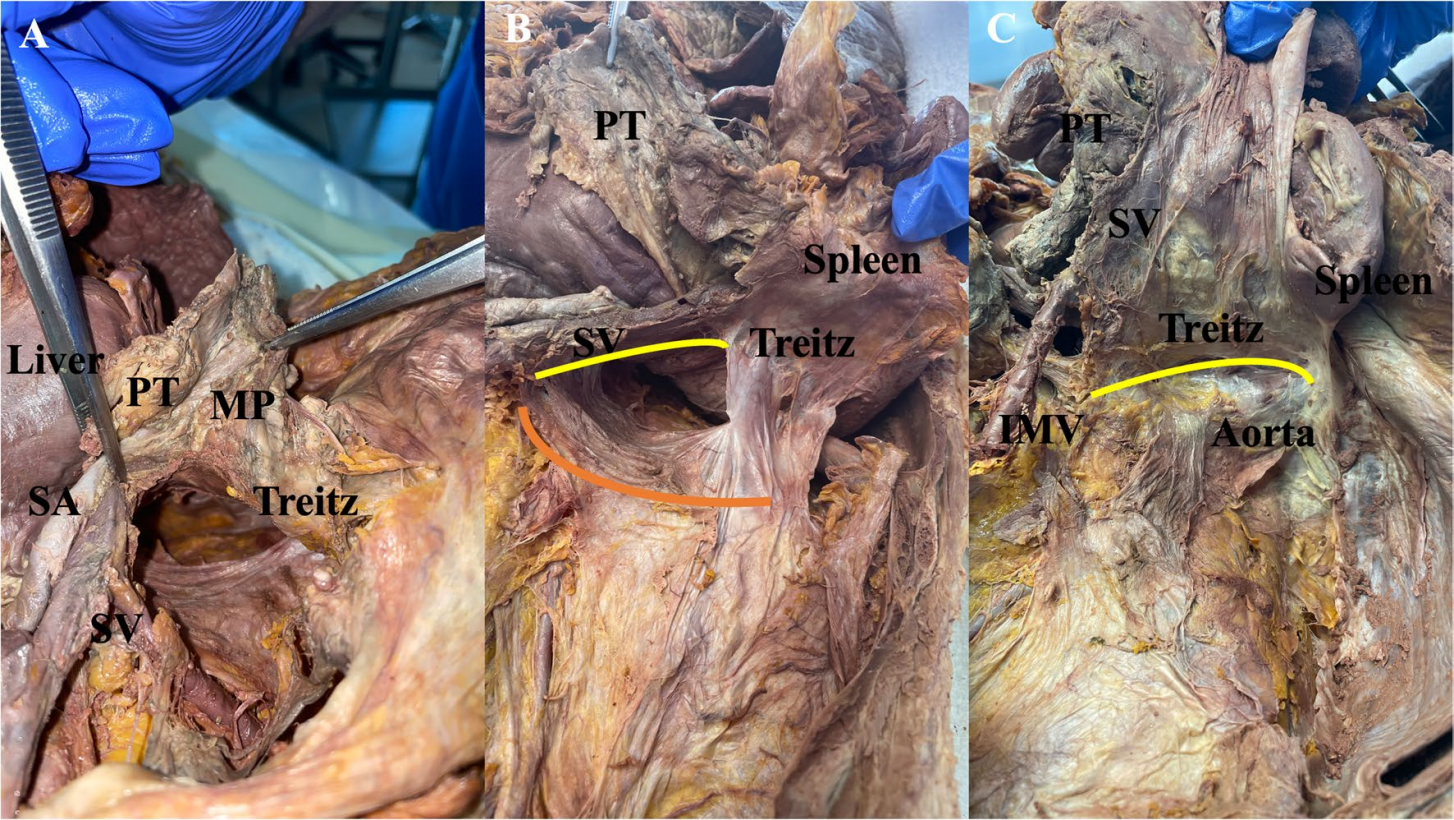
Picture series demonstrating anatomic preparations and dissections to visualize the peripancreatic fascial system of the distal pancreas. Note the embedded splenic vasculature in the fascial envelope, which can be identified as a single compartment. **A** Picture taken from caudo-cranial body donors left side. The splenic vasculature has already been mobilized from the posterior adhesions. The fascial covering (Treitz fascia) beneath the spenic vasculature encompasses both the vasculature, mesopancreas and the pancreatic tissue as a single anatomic compartment. **B** Picture taken from caudo-cranial, body donors left side. The distal pancreatic anatomical compartment (yellow line) is visualized together with the spleen and the Treitz fascia is separated from the Gerota fascia (orange line). **C** picture taken from caudo-cranial, body donors left side. The distal pancreatic compartment is mobilized together with the treitz fascia and the spleen towards the aorta, completing mobilization of the resectate. (*IMV* inferior mesenteric vein, *MP* mesopancreas, *PT* pancreatic tissue, *SA* splenic artery, *SV* splenic vein)

In order to elucidate the anatomy of the peripancreatic fascial system, which has been rarely described so far, surgical dissections under anatomic supervision (W.N.) have been performed on two consecutive cadavers from the Anatomic Institute I of the University of Erlangen-Nuremberg (Fig. 2). To revise the anatomic topography of the fusion fascia we performed these dissections in formaldehyde fixated body donors, which are well suited to identify the fascial residues.

### Histopathological analysis

**Fig. 3:**
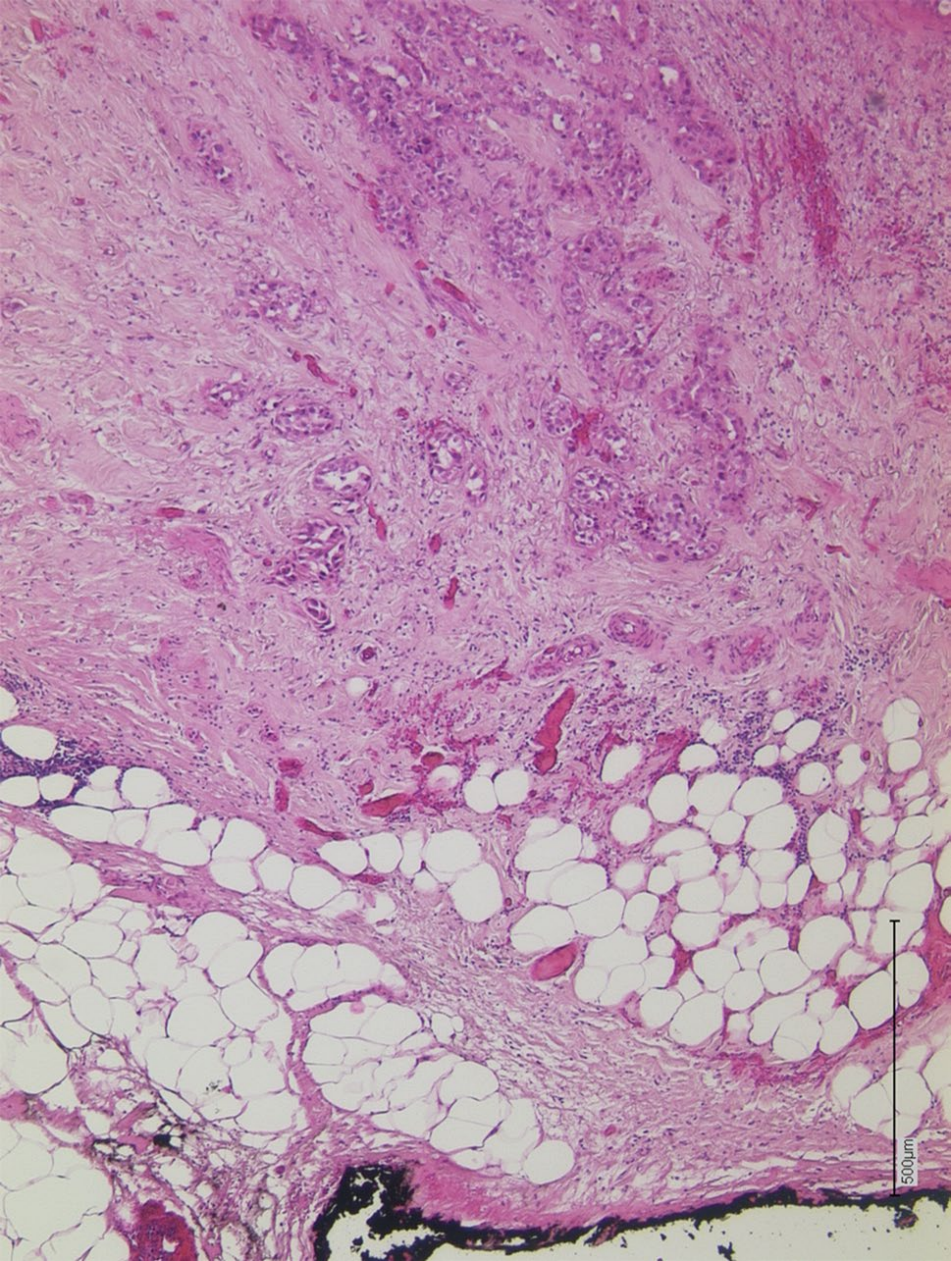
H&E section of a pancreatic ductal adenocarcinoma of the pancreatic tail. Tumor glands (top of the picture) are approaching the dorsal resection margin (arrow) (black ink, bottom of the picture). The resection margin consists of fatty tissue and fascia-like connective tissue. Since a fascial envelope is microscopically visible, a possible inclusion for histopathological quality assessment of surgical resection is possible (i.e. Mercury grading). Pathological staging results: pT2N1(2/25, ECE +)L1V0Pn1G3R0CRM–. (H&E, 50x)

The CRM evaluation was implemented at the University Hospital of Duesseldorf in September 2015. The oral/aboral duodenal, bile duct and pancreatic neck resection margin, as well as the dorsal resection margin and, if applicable, portal vein specimen, were examined according to the LEEPPs pathological protocol. Additionally, the mesopancreatic adipose tissue was histopathologically evaluated for cancerous infiltration (Fig. 3). Histopathological slides originating before 2015 were re-visited by a pathologist experienced in the hepatopancreaticobiliary system, and if sufficient slides were available, a CRM status with evaluation of the mesopancreatic fat was established. This included the evaluation not only of the dorsal, but also ventral and medial CRM. In addition, the “1-mm rule” was implemented: a minimum margin clearance of 1 mm defined R0CRM–, whereas margin clearances between 0–1 mm were judged as R0CRM + (Fig. 3) [29].

### Tumor board and follow-up

All patients were evaluated and discussed in an interdisciplinary tumor board pre- and postoperatively. Follow-up examinations were routinely performed every 3 months for the first 2 years, followed by every 6 months thereafter, including physical examination, blood chemistry, computed tomography of the chest and abdomen and abdominal ultrasound. Patients with suspicious metachronous masses were discussed in our tumor board for further therapy. If follow-up procedures were performed at other institutions, survival records of patients were gathered from the legal registration office.

### Statistics

The Mann–Whitney U test was used to examine numerical data and to correlate between clinico-pathological variables. For categorical data, the chi-square or fisher exact tests were applied. Overall survival (OS) and disease free survival (DFS) were included as outcome measures. OS was determined as the period from the date of surgery until the date of death or last follow-up. DFS determined the period between the date of surgery until the diagnosis of local recurrence. Kaplan–Meier curves were generated and analyzed using the log-rank (Mantel Cox) test, and hazard ratios (HR) with 95% confidence intervals (CI) were estimated. To perform a multivariate survival analysis, significant variables from the univariate analysis were included into a forward logistic regression analysis. Analyses were performed using SPSS statistics for Windows (version 26.0; SPSS, Inc., Chicago, IL, USA). Statistical significance was defined as *p* < *0.05*.

## Results

### Anatomical data

After dissecting the pancreatic tissue above the crossing of the superior mesenteric vein, the confluence of the splenic vein is visualized from the superior mesenteric vein just before its entry. The splenic artery is visualized from the celiac trunk. Both vessels are visualized, and their pathway is detected in the embedded retropancreatic fat, covered by a fibrous sheet (Fig. 2A). This fibrous sheet was followed until the splenic attachments from the lateral borders which continue medio-caudally and latero-caudally to the mesenteric origin of the transvers colon and descending colon attachments (Gerato and Toldts fascia) (Fig. 2B). Thus, the anatomic compartment of the pancreatic body and tail which can be dissected and separated from the anatomic compartment of the descending colon is embedded by the same fibrous sheet from dorsally (Fig. 2C). The historic descriptions of the Treitz fascia and Toldts fascia [12, 21, 30, 31] were confirmed, which underlines the existence of a peripancreatic compartment anatomy and the possibility to define splenopancreatectomy by anatomic hallmarks.

### Demographic data

Table 1 summarizes clinico-pathological characteristics of the cohort. A total of 295 consecutively treated patients met the inclusion criteria mentioned above and were included for further analysis (232 hPDAC patients and 61 dPDAC patients) (Table 1). The median age of all patients at the time of surgery was 69 years (range 17–90 years) (Table 1). The most notable difference was the median tumor size in dPDAC and hPDAC patients. The median tumor size with 40.0 mm in dPDAC patients was significantly larger than the median tumor size in hPDAC patients (median 26.0 mm) (*p* < *0.001*) (Table 1). Subsequently, T-stage was significantly higher in dPDAC patients when compared to hPDAC patients (*p* < *0.001)* (Table 1).

**Table 1:** Demographic table of all 295 studied patients. Staging is revised to the 8th edition of the UICC TNM classification of malignant tumors. Compared to hPDAC patients, dPDAC patients were diagnosed with a rather advanced tumor stage (T-, N and M-status). Resection status and rate of MP infiltration was similar between both groups Statistical significance was calculated by Mann–Whitney U test and chi squared test. ** indicates a *p*-value ≤ *0.01*; * indicates a *p-*value ≤ *0.05*

	hPDAC *n* = 232		dPDAC *n* = 63		*p-*value
Age in years					
Median (range)	69.0 (41–90)		68.5 (17–86)		0.267
Gender	*n*	%	*n*	%	0.577
Male	130	56.0	38	60.3	
Female	102	44.0	25	39.7	
T-stage					**< 0.001**
T1	2	8.6	4	6.3	
T2	126	54.3	18	28.6	
T3	80	34.5	37	58.7	
T4	6	2.6	4	6.3	
Tumorsize (mm)					**< 0.001**
Median (range)	26.5 (2.0–60.0)		40.0 (5.0–150.0)		
*N*-stage					**< 0.001**
N0	38	16.4	24	38.1	
N1	106	45.7	26	41.3	
N2	88	37.9	13	20.6	
Grading					0.459
G1/G2	135	58.2	39	61.9	
G3	97	41.8	24	38.1	
Pn					0.127
Pn0	54	23.3	18	28.6	
Pn1	178	76.7	45	71.4	
L					0.179
L0	122	52.6	38	60.3	
L1	110	47.4	25	39.7	
V					0.902
V0	174	75.0	48	76.1	
V1	58	25.0	15	23.9	
R-status					0.453
R0(CRM–)	114	49.1	33	52.4	
R1/R0(CRM +)	118	50.8	30	47.6	
MP Infiltration					0.497
MP positive	177	76.3	47	74.6	
MP negative	55	23.7	16	25.4	

*CRM* Circumferential resection margin; *L* Lymphatic invasion; *MP* Mesopancreatic; *Pn* Perineural invasion; *V* Venous invasion

### Histopathological results

#### Resection status

Following the 1 mm-rule, CRM assessment was available in all patients (*n* = 295). 146 patients (49.8%) were staged as R0(CRM–), whereas the remaining 147 patients (50.2%) had tumor infiltration into the 1 mm resection margin R0(CRM +) or were R1 resected (Table 1). Distribution of clinicopathological variables in completely resected and R1/R0(CRM +) resected dPDAC patients was tabulated separately (Table 2). Resection margin was not affected by the strictly size based T-stage (*p* = *0.801*). In dPDAC patients, all clinicopathological variables, except Pn-, V- and L-status, were homogenously distributed across resection margin status (Table 2).

**Table 2:** Correlation analysis of patients stratified according to resection margin status, *n* = 63. In dPDAC patients clinicopathological variables were homogenously distributed across resection margin status (TNM). Only Pn1, V1 and L1 posivitiy correlated with an increased rate of R1/R0CRM + resection. Statistical significance was calculated by chi squared test. ** indicates a *p-*value ≤ *0.01*; * indicates a *p-*value ≤ *0.05*

	R1/R0CRM + *n* = 30		R0CRM – *n* = 33		*p-*value
Age in years					
Median (range)	67.5 (47–90)		69.0 (41–88)		
Gender	*n*	%	*n*	%	0.641
Female	11	36.7	42.4	42.4	
Male	19	63.3	57.6	57.6	
T-stage					0.801
T1 and T2	10	33.3	12	36.4	
T3 and T4	20	66.7	21	63.6	
*N*-stage					0.064
N0	7	23.3	17	51.5	
N1	16	53.3	10	30.3	
N2	7	23.3	6	18.2	
Grading					0.213
G1/G2	16	53.3	23	68.8	
G3	14	46.7	10	31.3	
Pn					**0.001**
Pn0	3	10.0	15	46.9	
Pn1	27	90.0	18	53.1	
L					**0.043**
L0	14	46.7	24	71.9	
L1	16	53.3	9	28.1	
V					**0.026**
V0	19	63.3	29	87.5	
V1	11	36.7	4	12.5	

*CRM* Circumferential resection margin; *hep* Hepatic; *L* Lymphatic invasion; *Pn* Perineural invasion; *V* Venous invasion

#### Mesopancreatic evaluation

In all 295 patients (Table 1), FFPE specimens were available for re-evaluation of the posterior mesopancreatic fat tissue. The rate of MP infiltration was similar in dPDAC and hPDAC patients (*p* = *0.497*) (Table 1). Correlation analysis between clinicopathological variables and MP status was performed in dPDAC patients separately (Table 3). MP + patients were significantly more prone to an advanced T-stage when compared to MP– patients (*p* = *0.038*). All other studied clinicopathological variables were homogenously distributed across MP infiltration status (Table 3). R0CRM– resection rate in MP– patients was 62.5% and thus higher than in MP + patients (48.9%), yet did not reach statistical significance (*p* = *0.348*).

**Table 3:** Correlation analysis of dPDAC patients stratified according to positive and negative mesopancreatic infiltration, *n* = 63. dPDAC patients with an advanced T-stage were more prone to MP + status. There was a homogenous distribution of all other clinico-pathological variables in dPDAC patients when stratified according to MP status. Statistical significance was calculated by chi squared test. ** indicates a *p-*value ≤ *0.01*; * indicates a *p-*value ≤ *0.05*

	MP – *n* = 16		MP + *n* = 47		*p-*value
Age in years					
Median (range)	67.5 (47–90)		69.0 (41–88)		
Gender	*n*	%	*n*	%	0.836
Female	6	37.5	19	40.4	
Male	10	62.5	28	59.6	
T-stage					**0.038**
T1 and T2	9	56.3	13	27.7	
T3 and T4	7	43.8	34	72.3	
*N*-stage					0.306
N0	8	50.0	16	34.0	
N1	4	25.0	22	46.8	
N2	4	25.0	9	19.1	
Grading					0.908
G1/G2	10	62.5	29	60.9	
G3	6	37.5	18	39.1	
Pn					0.386
Pn0	6	37.5	12	26.1	
Pn1	10	62.5	35	73.9	
L					0.360
L0	8	50.0	30	63.0	
L1	8	50.0	17	37.0	
V					0.555
V0	13	81.3	35	73.9	
V1	3	18.8	12	26.1	
R-status					0.348
R0CRM–	10	62.5	23	48.9	
R1/R0CRM +	6	37.5	24	51.1	

*CRM* Circumferential resection margin; *hep* Hepatic; *L* Lymphatic invasion; *Pn* Perineural invasion; *V* Venous invasion

### Survival analysis in dPDAC patients

Three out of 63 patients deceased during the first 90 postoperative days (Clavien-Dindo V; 90-day mortality rate: 4.7%)**.** Follow up data of all 63 patients was obtained using official records from the registration office. The median OS of the 63 M0 resected patients was 41.00 months (95%CI: 19.30 – 58.70 months).

**Fig. 4:**
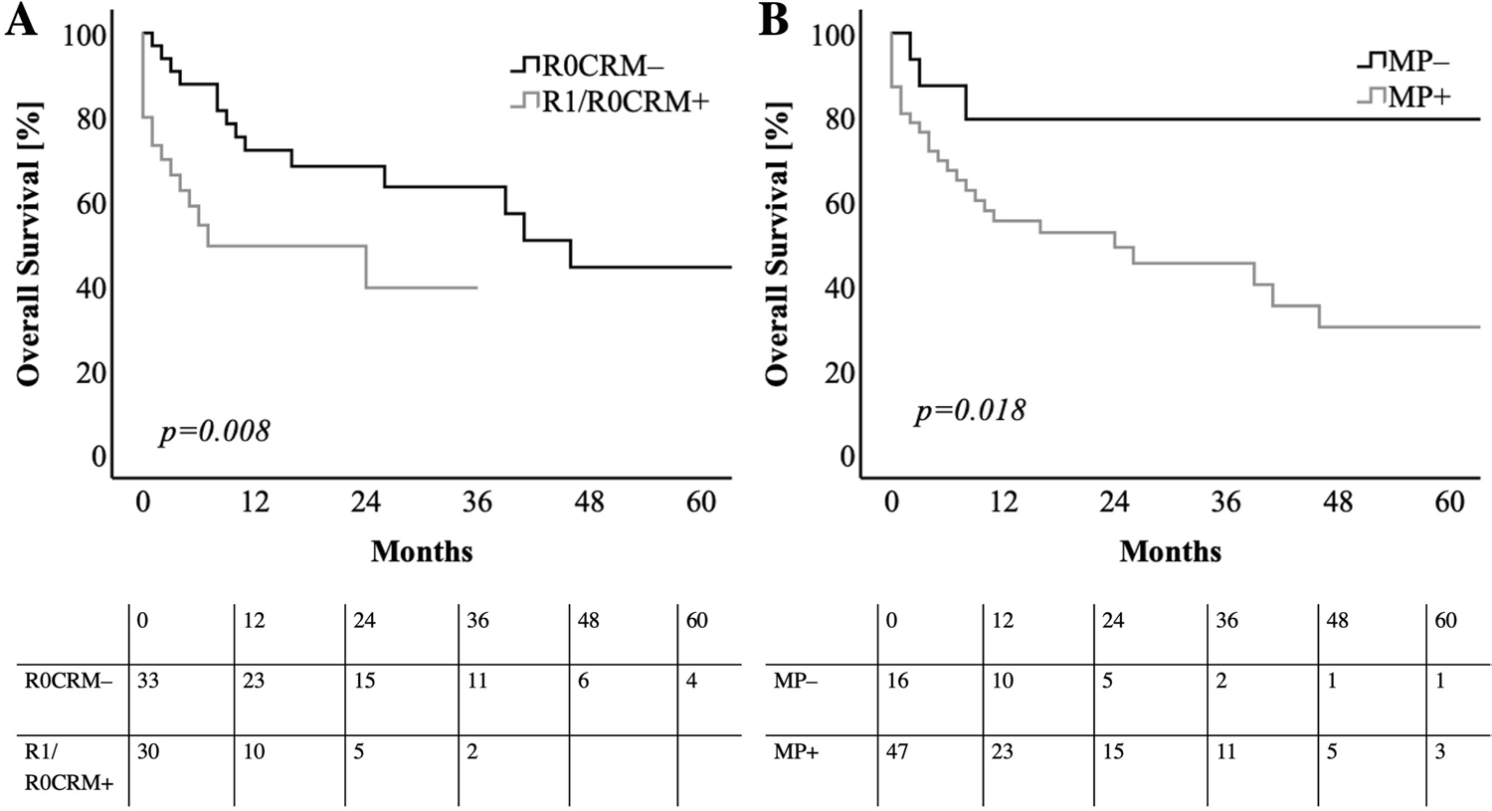
Kaplan–Meier curves for **A** overall survival in correlation with positive and negative resection status in CRM evaluated dPDAC patients. dPDAC patients after R0CRM– resection showed a significant improvement in overall survival. **B** overall survival of dPDAC patients stratified according to the mesopancreatic (MP) infiltration status. dPDAC patients without infiltration of the MP showed surprisingly a major benefit in overall survival (median 60 months) when compared to patients with MP infiltration. *MP* = *mesopancreatic.* Log rank test was used to test for significance. *p-*value ≤ *0.05* is regarded as significant

The median OS was stratified according to the resection status. In the margin negative patients (R0CRM–; *n* = 33) the median OS (44.51 months, 95%CI: 33.93–58.07 months) was significantly longer, compared to the margin positive patients (R1/R0CRM + ; *n* = 30) (median: 11.57 months, 95%CI: 1.00–25.93 months) (*p* = *0.008)* (Table 4, Fig. 4A). We observed similar findings when dPDAC patients were stratified according to the MP status. In patients without a MP infiltration (*n* = 16 patients), OS was significantly prolonged, compared to patients with MP infiltration. The median OS was 60.00 months in the MP– group vs. 28.33 months in the MP + group *(p* = *0.018)* (Table 4, Fig. 4B). Of all studied variables, positive resection margin and MP infiltration status remained prognostic parameters in univariate survival analysis (Table 4). In multivariate survival analysis MP infiltration status remained an independent prognostic factor for poor overall survival (Table 4).

**Table 4:** Univariate and multivariate survival analyses for overall survival of resected dPDAC patients; *n* = 63. Analyses were performed by log-Rank test and cox logistic forward regression. *p-*value ≤ *0.05* is considered statistically significant

Univariate analysis				
	*p-*value			
Median age (< vs. > median)	0.134			
Sex (female vs. male)	0.496			
T-stage (T1/T2 vs. T3/T4)	0.503			
*N*-stage (N0 vs. N1/N2)	0.190			
Grading (G1/G2 vs. G3)	0.068			
Pn (Pn0 vs. Pn1)	0.081			
L (L0 vs. L1)	0.250			
V (V0 vs. V1)	0.298			
R-status (R0CRM–) vs. R1/R0CRM +)	**0.008**			
MP-status (MP + vs. MP–)	**0.018**			
Multivariate analysis				
		*p-*value	HR	95%CI
MP-status (MP– vs. MP +)		**0.030**	9.49	(1.25–72.17)

*CI* Confidence interval; *CRM* Circumferential resection margin; *HR* Hazard ratio; *L*: Lymphatic invasion; *MP* Mesopancreas; *Pn* Perineural invasion; *V* Venous invasion

### Disease free survival and local recurrence in dPDAC patients

**Fig. 5:**
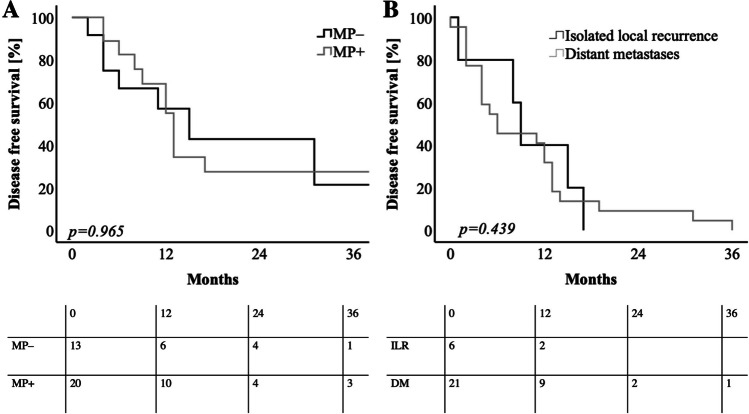
Kaplan–Meier curves for **A** disease free survival in correlation with positive and negative mesopancreatic (MP) infiltration status in CRM evaluated dPDAC patients. dPDAC patients with MP– infiltration showed a similar disease-free survival compared to dPDAC patients with MP + infiltration. **B** disease survival of dPDAC patients stratified according to location of diagnosed diagnose relapse. dPDAC patients with diagnosed isolated local recurrence (ILR) showed a similar DFS when compared to dPDAC patients with diagnosed distant metastases (DM). Log rank test was used to test for significance. *p-*value ≤ *0.05* is regarded as significant

Detailed follow up examinations with location of metastases were registered in 33 patients. Out of the 33 patients, 6 patients were diagnosed with local recurrence (Table 5) (R0CRM– *n* = 1; R1/R0CRM + *n* = 5). Thus, dPDAC patients receiving margin negative resections (R0CRM–) had a significantly lower rate of local recurrence when compared to patients with insufficient margin clearance (*p* = *0.002*) (Table 5). The median DFS was 13 months (95%CI: 11.50–14.49). dPDAC were stratified according to MP infiltration status. All dPDAC patients independent of the MP infiltration status had a similar DFS (MP– dPDAC patients median DFS 15.00 months 95%CI: 5.93–24.07 months vs. MP + dPDAC patients median DFS 13.00 months 95%CI: 11.83–14.17 months) (*p* = *0.965*) (Fig. 5A). Out of the 33 patients, 27 patients were diagnosed with disease relapse. DFS was computed in the 27 patients stratified according to location of metachronous disease (local recurrence vs. distant metastases). Median DFS in dPDAC patients diagnosed with isolated local recurrence and those with distant metastases was similar (9 months (95%CI: 2.14–15.86 months and 6.00 months 95%CI: 1.00–14.55 months, repectively) (*p* = *0.439*) (Fig. 5B).

**Table 5:** Correlation analysis of follow-up diagnoses for metachronous disease in 33 dPDAC patients. Patients after margin negative resections were diagnosed with a significantly lower rate of local recurrence. Statistical significance was calculated by chi squared test and log-Rank test. ** indicates a *p-*value ≤ *0.01*; * indicates a *p-*value ≤ *0.05*

dPDAC	Local recurrence *n* (%)	*p-*value
R0CRM – *n* = 19	1 (5.2)	**0.002**
R1 or R0CRM + *n* = 15	5 (33.3)

## Discussion

By incorporating the CRM into pathological evaluation, about 80% of historic pancreatic resections showed microscopic tumor residues at the surgical margins [32]. This resonates with the reported high rate of mesopancreatic fat infiltration [7], concluding that mesopancreatic excision (MPE) presumably increases the rate of true R0 resections (R0CRM–) in patients with PDACs of the pancreatic head [7, 24, 25].

Our anatomical preparations on human cadavers revealed the Treitz fascia of the pancreatic body and tail embedding both the organ, the mesopancreas and the splenic vasculature. We were also able to distinguish the Treitz fascia from the Gerota fascia, which underlines the existence of a compartment anatomy of the distal pancreas and a presumed embryo-anatomic justification of splenopancreatectomy [17]. We furthermore identified this fascia as a medio-cranial extension of the Toldt fascia, which is in line with known anatomical descriptions [12, 30, 31]. Distal splenopancreatectomy ensures a safe transection site for the dorsal pancreatic compartment [18]. Dorsal limitations of this transection is therefore the cancerous infiltration of the kidney or its vessels rather than the inferior caval vein/abdominal aorta, as in hPDAC patients.

Strasberg et al. described the first standardized resection technique for distal PDACs [17] and introduced the first series after the RAMPS procedure [18]. 73% of his patients had a tumor infiltration outside the pancreas presumably into the mesopancreas [18], however, the relevance of this pancreas-extending growth was not further investigated in the current literature. Anatomical hallmarks, such as the mesopancreas, have not been incorporated in the current discussions investigating the superiority between the conventional, anterior and posterior RAMPS [19, 20]. This could substantiate the heterogeneity in oncological outcome between the three approaches for splenopancreatectomy because of stratification bias and underestimated, histopathologically not assessed, tumor growth. Our data shows that the infiltration status of the mesopancreas is paramount for tumor clearance and could serve as a stratification variable for resection status and hence resectability. It further could serve as a justification parameter to simply standardized splenopancreatectomy for dPDAC patients in guidelines, making spleen preserving techniques in pancreatic malignancies obsolete and unjustified.

In this unicentric series, a positive MP infiltration status failed to directly correlate with a positive resection margin (R1/R0CRM +). One of the reasons could be the small cohort size of dPDAC patients, due to the lower incidence rate of dPDACs compared to hPDACs. A positive infiltration status of the mesopancreas could again only be predictive for incomplete resection, if a spleen-preserving approach, which many centers still perform in selected patients [14, 33], was compared to patients after splenopancreatectomy. Another reason could be the easier accessible anatomical topography of the pancreatic body/tail when compared to the pancreatic head. The anchor point of the mesopancreas lies around the superior mesenteric artery. During pancreatoduodenectomy for hPDACs, the close vicinity of an infiltrated mesopancreas to the SMA makes a safe transection around the SMA more challenging. Similar anatomic bottlenecks do not exist for oncological distal splenopancreatectomy. The splenic vasculature, which encompasses the dorsal resection margin (and the mesopancreas), is simultaneously removed, allowing a safe margin clearance at the dorsal resection margin [17]. Survival analysis in PDAC patients was already thoroughly studied in the past and there is a uniform consensus that R0CRM– resection is an important, surgically dependent, survival factor [34]. Surgery and perioperative management, as well as pathological re-evaluation, were performed by the same team during the entire study period on a consecutive patient cohort, limiting selection bias. In our study we confirmed R0CRM– status to be an important prognostic factor for prolonged survival.

In our cohort of dPDAC patients, MP + status was significantly more prevalent in patients with an advanced T-stage (T3/T4). All other staging variables were homogenously distributed across dPDAC patients stratified for MP-status. We previously observed a similar pattern in hPDAC patients (Table iii and iv, supplemental [35]). Yet we cannot explain why the MP status is equally affected in PDAC patients independent of tumor location and staging. Presumably genetic and epigenetic tumor analysis in a cohort of PDAC patients, stratified according to the MP status, could reveal more insight into this unusual tissue microenvironement. MP infiltration status was a stronger predictor for poor overall survival compared to all other studied variables including resection margin status. Previously, Demir et al. reported that CRM implemented resection status stratified survival outcome more obvious in hPDAC patients when compared to dPDAC patients, which is further underlined by our results [36]. Presumably, mesopancreatic infiltration status is a more predictive factor for survival in dPDAC patients.

This is the first study elucidating both the implemented pathological CRM and the evaluation of the mesopancreas in dPDAC patients. Our results suggest that anatomical hallmarks need to be considered during distal splenopancreatectomies, independent of the surgical approach [20]. By considering the (1) high rates of mesopancreatic fat infiltration found in dPDAC patients, (2) the anatomic proximity of the splenic vasculature to the mesopancreas and (3) the same fascial envelope compassing both structures, we conclude that mesopancreatic excision and thus splenopancreatectomy is merited in dPDAC.

## Conclusion

This study provides histopathological evidence that the mesopancreas is infiltrated in patients with PDACs of the pancreatic body and tail. It is the first study comparing the mesopancreatic invasion in PDACs arising from the pancreatic body/tail to those of the pancreatic head. The established local tumor control following MPE in our cohort justifies this embryologic derived surgical approach. Distal splenopancreatectomy should be employed in all dPDACs. Further multicentric studies are needed to validate our results with a greater patient cohort.

## Data Availability

The datasets used and/or analyzed during the current study are available from the corresponding author on reasonable request.

## Abbreviations

CHA: Common hepatic artery
CRM: Circumferential resection margin
dPDAC: Distal ductal adenocarcinoma of the pancreas (body/tail)
DM: Distant metastases
FFPE: Formalin fixed paraffin embedded
hPDAC: Pancreatic head ductal adenocarcinoma
ILR: Isolated local recurrence
IMV: Inferior mesenteric vein
L: Lymphatic invasion
LEEPP: Leeds Pathology Protocol
MP: Mesopancreatic
MPE: Mesopancreatic excision
PD: Pancreatoduodenectomy
Pn: Perineural invasion
PV/SMV: Portal/superior mesenteric vein
SA: Splenic artery
SMA: Superior mesenteric artery
SV: Splenic vein
UICC: Union international contre le cancer
V: Venous invasion
